# PROTOCOL: The effects of cannabis liberalization laws on health, safety, and socioeconomic outcomes: An evidence and gap map

**DOI:** 10.1002/cl2.1137

**Published:** 2021-01-13

**Authors:** Eric L. Sevigny, Rosalie L. Pacula, Ariel M. Aloe, Danye N. Medhin, Jared Greathouse

**Affiliations:** ^1^ Department of Criminal Justice and Criminology, Andrew Young School of Policy Studies Georgia State University Atlanta Georgia USA; ^2^ Sol Price School of Public Policy and Schaeffer Center for Health Policy & Economics University of Southern California Los Angeles California USA; ^3^ College of Education University of Iowa Iowa City Iowa USA; ^4^ Department of Political Science Georgia State University Atlanta Georgia USA

## 1 BACKGROUND

### 1.1 The problem, condition, or issue

Cannabis is one of the most commonly used psychoactive substances globally, with an estimated 3.9% of the world's population aged 15–64 reporting past‐year use (United Nations, 2020). Many world regions report higher annual prevalence rates (e.g., North America, 14.6%; Australasia, 10.6%; Western; and Central Europe, 7.8%), with certain countries in these regions documenting significant increases in cannabis use over the last decade (United Nations, 2020). Cannabis dependence accounts for a small fraction (5.5%) of the overall global burden of disease attributable to alcohol and drugs, but this burden commonly surpasses that of amphetamines in world regions with high rates of cannabis use (Degenhardt et al., 2013).

Against this backdrop, many countries and US states have liberalized their cannabis laws over the past 25 years (Decorte et al., 2020). Between 1996 and year‐end 2019, 33 US states plus the District of Columbia enacted medical marijuana laws granting authorized patients legal access to cannabis.^1^ Moreover, since 2012, 11 states and the District of Columbia passed recreational marijuana laws legalizing adult use, including retail sales in nine states. These developments have led to a patchwork of state laws regulating access to cannabis through a variety of supply mechanisms, with state legislatures and citizen initiatives continuing to spur both new laws and amendments to existing laws (Chapman et al., 2016; Hoffmann & Weber, 2010; Klitzner et al., 2017; Williams et al., 2016). Indeed, voters in five US states will consider recreational or medical cannabis initiatives during the 2020 election. Dozens of other countries have also expanded legal access to cannabis under a variety of regulatory models, including decriminalization of home cultivation and the establishment of “cannabis social clubs” (Belackova et al., 2020; Decorte et al., 2017, 2020; Fischer et al., 2015; Rehm et al., 2019). Perhaps most notably, Uruguay became the first country to legalize recreational cannabis in 2013 (Queirolo, 2020), followed by Canada in 2018 (Fischer et al., 2020).

The empirical literature examining the effects of cannabis laws and policies is interdisciplinary and diverse. New research appears almost weekly, with studies examining a wide range of health, safety, and socioeconomic outcomes. Health outcomes measure physical and mental well‐being or disease including cardiovascular disease (Abouk & Adams, 2018), opioid overdose (Chan et al., 2020), and suicide (Anderson et al., 2014; Chan et al., 2020). Safety outcomes measure security or risk of harm including crime (Morris et al., 2014), impaired driving (Sevigny, 2018), and vehicular accidents (Salomonsen‐Sautel et al., 2014). Socioeconomic outcomes capture social and economic metrics including property values (Burkhardt & Flyr, 2019), labor supply (Nicholas & Maclean, 2019), and educational attainment (Plunk et al., 2016). To date, no concerted efforts have fully scoped this varied and growing literature. Consequently, stakeholders have an incomplete understanding of the effects of cannabis liberalization laws. This EGM aims to fill this gap by collecting and summarizing the available evidence for decision‐makers and identifying opportunities for future knowledge generation.

### 1.2 Scope of the EGM

This EGM summarizes research investigating the effects of cannabis liberalization reforms on health, safety, and socioeconomic outcomes internationally. This purview encompasses statutory and regulatory provisions governing legal access to cannabis among both specific (e.g., patients with certain medical conditions) and general (e.g., adults 21 and older) populations. For outcomes, the EGM will capture the array of health, safety, and socioeconomic outcomes reported in the literature.

### 1.3 Conceptual framework of the EGM

**Figure 1:**
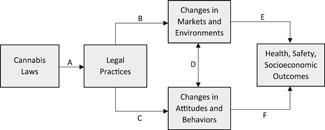
Conceptual framework for understanding the effects of cannabis liberalization laws on health, safety, and socioeconomic outcomes

This EGM will document how cannabis law reforms affect health, safety, and socioeconomic outcomes across different target populations (e.g., patients, adults). The conceptual framework presented in Figure 1, which draws from legal epidemiology (Burris et al., 2013, 2016), depicts a causal chain between cannabis laws and associated outcomes. Cannabis laws reflect the “law on the books” as determined by statutes and regulations. Assessing policy impacts also requires understanding implementation, or the “law on the streets.” Path A captures these on‐the‐ground legal practices that may not be reflected in statute but still impact outcomes in meaningful ways.

Paths B and C depict the effects of laws and legal practices on markets/environments (e.g., cannabis prices and potency, drug arrests) and attitudes/behaviors (e.g., frequency of cannabis use, modes of consumption). Bilateral Path D recognizes that markets/environments and attitudes/behaviors may influence each other, such as the plausible inverse association between cannabis potency and frequency of use due to longer‐lasting periods of intoxication. Finally, paths E and F capture population‐level health, safety, and socioeconomic impacts as a consequence of intermediate changes in markets/environments and attitudes/behaviors.

Although understanding the direct effects of law on health, safety, and socioeconomic outcomes is often a primary concern, highlighting these links informs study inclusion and coding decisions. The framework differentiates studies examining, for instance, legal allowances for recreational dispensaries (cannabis law) from those examining geographic variation in recreational dispensary density (legal practice). The conceptual framework allows coders to capture these important differences in the EGM. Likewise, intermediate market/environment and attitude/behavior outcomes are conceptualized along the causal chain in the framework, as they can enhance or mitigate health, safety, and socioeconomic consequences, and are thus worthy of documentation and study in their own right. This is particularly true when insufficient time has passed to allow a complete and valid assessment of longer‐term outcomes (e.g., cardiovascular or respiratory health). In short, the conceptual framework guides our understanding of where in the causal chain each study's interventions and outcomes are situated, and how they might be incorporated into the EGM.

### 1.4 Why it is important to develop the EGM

Despite a quarter‐century or more of cannabis liberalization reforms across the globe, no EGM exists to summarize the available evidence and knowledge gaps in this policy space. The development of this EGM will fill this need. Importantly, it will document the heterogeneity in target populations (Pacula & Smart, 2017), variation in policy design and implementation (Klieger et al., 2017; Klitzner et al., 2017), and diversity of outcomes across multiple sectors (Fischer et al., 2018; Maslov et al., 2016). Moreover, current research increasingly employs complex methods and statistical models (Choo & Emery, 2017; Hunt & Miles, 2015). Mapping this knowledge base is therefore needed to (i) provide decision‐makers with a catalogue of quality evidence on cannabis liberalization reforms and (ii) identify knowledge gaps for prioritizing new primary research and systematic reviews.

### 1.5 Existing EGMs and/or relevant systematic reviews

To our knowledge, this review will provide the first comprehensive EGM of cannabis liberalization laws and associated policy outcomes. While there are now several published systematic narrative reviews (Hunt & Miles, 2015; Leung et al., 2018; Smart & Pacula, 2019; Vyas et al., 2018) and meta‐analyses (Melchior et al., 2019; Sarvet et al., 2018) in this policy space, they primarily assess the effects of medical and recreational cannabis laws on drug use outcomes among US youth and adults. Researchers have examined a much broader and arguably more salient set of policy outcomes than just cannabis use (Sznitman & Zolotov, 2015; Waddell & Wilson, 2017). Recent conceptual mappings across varied national contexts have identified dozens of performance indicators in the public health, community safety, economic, and children and youth sectors that are relevant for understanding the full scope of cannabis policy impacts (Campeny et al., 2020; Fischer et al., 2018; Maslov et al., 2016).

## 2 OBJECTIVES

The proposed EGM will seek to answer several questions. What is the extant evidence‐base on cannabis liberalization policies, and how does this evidence vary across policy types (i.e., medicalization, legalization, decriminalization)? What outcomes are predominant in the available literature? What gaps exist in the literature, and are there clusters of studies that can support future systematic reviews and meta‐analyses? How do studies vary by target population, geographic focus, and analytic methods? Along these lines, the objectives of this EGM are to:

1. Develop a comprehensive intervention‐outcome framework of cannabis liberalization laws and related health, safety, and socioeconomic outcomes.
2. Map relevant systematic reviews and primary studies within this framework.
3. Summarize the intervention, outcomes, context, study design, study quality, and main findings of included studies.
4. Highlight clusters of studies that can support further systematic review and meta‐analysis, and document gaps where further primary research and resources are needed.

## 3 METHODOLOGY

### 3.1 Evidence and gap map: Definition and purpose

An EGM is a systematic review tool that provides a visual summary of strength of evidence and knowledge gaps within a particular policy domain (Miake‐Lye et al., 2016; O'Leary et al., 2017). EGMs are a recent addition to the systematic reviewer's toolkit (Da Silva et al., 2017; Saran & White, 2018). This EGM will be developed and populated according to the following steps: (i) develop a conceptually‐driven intervention‐outcome framework, (ii) define study inclusion criteria and establish literature search strategy following established guidelines, (iii) devise a coding instrument for data extraction and critical appraisal of studies, including brief summaries of main findings. Findings will be presented in a graphical and/or tabular formats to better inform the current status of evidence and inquiry, including assessments of study quality and gaps in knowledge (Lum et al., 2011; Snilstveit et al., 2016).

### 3.2 EGM framework

#### 3.2.1 Population

For this EGM, populations will be defined by subgroups that are reflected in the primary research and provide meaningful cross‐study aggregations. Target populations vary with the type of cannabis law. For instance, recreational cannabis laws apply to the general adult population (typically 21+), whereas medical cannabis laws apply more narrowly to individuals diagnosed with certain medical conditions (e.g., epilepsy, HIV, chronic pain). Because research in this area is often concerned with unintended consequences, such as illicit diversion of cannabis to youth, study populations are generally more encompassing than those defined by statute. Researchers in this area often account for other types of population heterogeneity, including age, gender, race/ethnicity, riskiness of use (e.g., heavy vs. casual users), and criminal justice involvement (Pacula & Smart, 2017). These various populations will serve as filters in the interactive EGM.

#### 3.2.2 Interventions

Interventions are a primary dimension of the EGM. Table 1 presents the specific cannabis liberalization policies covered by this EGM. The top level of the taxonomy captures the major type of cannabis law, including both comprehensive and limited (i.e., CBD/low‐THC) medical cannabis laws, recreational (i.e., adult access) cannabis laws, and decriminalization and industrial hemp laws that establish a licit supply of consumable cannabis. Regulatory domains and specific policy provisions in the taxonomy are drawn from previous legal mappings in this policy space (Bestrashniy & Winters, 2015; Chapman et al., 2016; Klieger et al., 2017; Klitzner et al., 2017; Pacula et al., 2014; Williams et al., 2016). Note that listed provisions are not relevant to each major law, as regulations concerning qualifying medical conditions, for instance, apply only to medical cannabis laws.

**Table 1:** Cannabis liberalization policy taxonomy

Domain	Provision
Major type of law	– Comprehensive medical cannabis law
	– Limited medical cannabis law
	– Recreational cannabis law
	– Decriminalization of cannabis cultivation
	– Industrial hemp law
User authorization provisions	– Registry/identification cards
	– Qualifying medical conditions
Medical professional provisions	– Professional certifications
	– Provider‐patient relationship
Caregiver provisions	– Registry/identification cards
	– Home cultivation
	– Plant/possession limits
	– Caregiver qualifications
	– Caregiver‐patient relationship
Personal supply provisions	– Home cultivation
	– Plant/possession limits
Social supply provisions	– Growing cooperatives/collectives
	– Cannabis social clubs
	– Plant/possession limits
Commercial supply provisions—production	– Commercial production
	– Registration/licensing
	– Zoning/density
	– Physical security
	– Workplace safety
	– Pesticide and fertilizer controls
	– Cannabis waste regulations
	– Production limits
Commercial supply provisions—retail	– Dispensaries/retail outlets
	– Cannabis cafes
	– Delivery services
	– Mail order services
	– Registration/licensing
	– Zoning/density
	– Physical security
	– Sales limits
Product provisions	– Product type restrictions
	– Testing requirements
	– Labeling/packaging
	– Product tracking
Marketing and advertising provisions	– Advertising regulations
	– Product display regulations
Financial provisions	– Taxes
	– Banking regulations

#### 3.2.3 Comparison

Typically, studies compare cannabis liberalization policies to status quo cannabis prohibition (i.e., “business as usual”). In some cases, the comparator may be an earlier adopted cannabis law. For example, a study may report the effect of recently adopted recreational cannabis laws against previously enacted medical cannabis laws. We will code these comparators to serve as filters in the interactive EGM.

#### 3.2.4 Outcomes

Outcomes are the second primary dimension of the EGM, which include intermediate (changes in markets and environments, changes in attitudes and behaviors) and final outcomes (health, safety, socioeconomic). Broadly, health outcomes measure physical and mental well‐being or disease, safety outcomes measure security or risk of harm, and socioeconomic outcomes capture community and fiscal impacts. Intermediate outcomes reflect changes in markets and environments (e.g., cannabis prices and potency, advertising, and marketing) and changes in attitudes and behaviors (e.g., attitudes toward marijuana, modes of consumption such as vaping), encompassing factors that shift the structural conditions and incentives impacting final outcomes. Table 2 identifies candidate intermediate and final outcomes to be coded. The listed outcomes draw from existing conceptual mappings of potential cannabis policy impacts (e.g., Campeny et al., 2020; Fischer et al., 2018; Maslov et al., 2016). The list will be updated based on studies included in the EGM. Along with expected positive impacts of these laws (e.g., reduced criminal justice costs, greater tax revenues), the purview of the EGM also encompasses the range of potential adverse or unintended outcomes (e.g., drugged driving and explosions/fires related to cannabis production).

**Table 2:** Intermediate and final marijuana policy outcomes

	Examples
*Intermediate outcomes*	
Changes in markets and environments	Arrests, prices, potency, diversion, marketing, product contamination
Changes in attitudes and behaviors	Attitudes toward substance use, prevalence of substance use, patterns and frequency of substance use, modes of consumption
*Final outcomes*	
Health impacts	Substance use disorders, treatment admissions, emergency care utilization, overdose, suicide, mental and physical health, neonatal and pediatric exposure
Safety impacts	Crime, organized crime, drugged driving, traffic accidents and fatalities, workplace safety, production‐related explosions, and fires
Socioeconomic impacts	Housing values, educational attainment, productivity, (un)employment, sick days, industry jobs, GDP, criminal justice costs, healthcare costs, tax revenues, drug tourism, environmental consequences

### 3.3 Criteria for including and excluding studies

#### 3.3.1 Interventions and outcomes

Interventions are eligible if they increase legal access to cannabis supply. This includes recreational cannabis laws and both comprehensive and limited product (i.e., CBD/low‐THC) medical cannabis laws. Only cannabis decriminalization policies that remove criminal penalties for small‐scale cannabis cultivation are eligible because they establish a legitimate (or tolerated) supply of cannabis. Lastly, we do not explicitly include industrial hemp laws that regulate and incentivize cannabis production for textiles, biofuel, and other commercial uses. However, these laws can open loopholes for sales of cannabis products meant ostensibly, albeit not legally, for human consumption (e.g., Carrieri et al., 2019). Consequently, we will include studies that examine whether the sale of cannabis products for human consumption increases as a result of these laws.

Some primary studies may investigate cannabis liberalization policies in various policy combinations (e.g., states with both medical *and* recreational cannabis laws). If the study combines eligible interventions, it will be included in the EGM and identified as a combined policy to avoid misleading comparisons with individually assessed policies. If the combined intervention includes any ineligible intervention (e.g., decriminalization of cannabis use), it will be excluded from the EGM.

In accordance with Figure 1, the EGM aims to capture all intermediate (markets/environments and attitudes/behaviors) and final (health, safety, and socioeconomic) outcomes, including those characterized as adverse. Thus, within this framework, we place no a priori exclusions on specific outcomes. Our purview is purposely broad in scope.

#### 3.3.2 Types of study designs

Eligible studies employ quasi‐experimental designs that control for confounding factors, have a relevant comparator, and/or assess change over time. Nominally, this includes cohort studies, difference‐in‐differences (DID), interrupted time series (ITS), propensity score analysis (PSA), regression discontinuity designs (RDD), synthetic control methods (SCM), and instrumental variables estimation (IVE). Cohort studies compare intervention and comparison conditions on the basis of exposure status in which subjects are not randomized or otherwise allocated by the researchers. DID designs are similar to cohort studies except that subjects are typically clustered and longitudinal preintervention data is required. ITS designs typically follow a single unit across time and asses change at or after the point of intervention (Reeves et al., 2017). PSA, RDD, SCM, and IVE are quasi‐experimental designs that address selection bias and other forms of endogeneity under specific statistical assumptions (Rockers et al., 2015). This EGM focuses on these types of observational designs because they have relatively high internal validity. Lastly, RCTs are eligible, although we do not anticipate finding this design as cannabis policies cannot be feasibly randomized. Studies that examine hypothetical policy scenarios using simulation or forecasting designs will not be included in the EGM, as we restrict our purview to studies analyzing real‐world data.

#### 3.3.3 Treatment of qualitative research

We do not include qualitative research in the EGM.

#### 3.3.4 Types of settings

Eligible settings include national or subnational jurisdictions that have legalized the supply of cannabis for medical or recreational purposes. There are no geographic or timeframe restrictions on eligibility.

#### 3.3.5 Types of participants

Studies examining impacts of cannabis liberalization policies focus on a heterogeneous mix of study participants given the varying target populations of these laws (e.g., medical patients, adults 21+). Study participants also extend to populations not directly targeted by these laws but who nevertheless are impacted by them (e.g., youth, unimpaired drivers). Study participants could even include family pets, as case research has documented increased likelihood of cannabis toxicosis in dogs in legal cannabis states (e.g., Meola et al., 2012). We, therefore, place no a priori restrictions on study participants to include this diversity of studies.

#### 3.3.6 Status of studies

Completed and ongoing research will be included in the EGM. For completed research, published and unpublished (e.g., dissertations, working papers, reports) studies will be included. Working papers or preliminary studies that are subsequently published will updated in the EGM as they become available.

### 3.4 Search strategy

Our search strategy engages academic databases, gray literature databases, researchers in the field, the authors' personal research libraries, and backward and forward citation searching of included studies. Database searching will employ both natural language and controlled vocabulary terms, queried using Boolean logic across three domains: (1) cannabis/marijuana, (2) policy change, (3) and quantitative methods (Glanville et al., 2017; Tompson & Belur, 2015). Specific search terms and logic, including the use of wildcards, are presented in Table 3. The search strategy will be amended as appropriate to accommodate less sophisticated search engines typical of gray literature databases. For each database, specific search terms, dates of search, and any additional delimiters (e.g., excluding newspaper records) will be recorded. We will also document any outreach efforts to researchers in the field. Studies written in English since 1970 will be eligible for the EGM. We target English‐only publications because not all members of the study team are fluent in other languages. The year 1970 captures all modern cannabis liberalization reform efforts.

**Table 3:** Search terms and boolean logic

Domain	Natural language^a^		Controlled vocabulary
1. Cannabis	cannabis OR marijuana OR hemp	OR	“cannabis” OR “cannabis edibles” OR “hemp” OR “marijuana” OR “medical marijuana”
AND			
2. Policy Change	commercializ* OR “cannabis social club*” OR cultivat* OR decrim* OR dispensar* OR grow* OR industr* OR law* OR legal* OR legislat* OR liberaliz* OR medical* OR polic* OR program* OR recreation* OR regulat* OR retail*	OR	“commercialization” OR “drug control” OR “drug laws & regulations” OR “drug legalization” OR “government policy” OR “government regulation” OR “government regulations” OR “law” & “legalization” OR “legislation” OR “legislation, drug” OR “legislation, medical” OR “liberalization” OR “marijuana dispensaries” OR “marijuana growing” OR “marijuana industry” OR “marijuana laws” OR “marijuana legalization” OR “medical marijuana ‐‐ law & legislation” OR “medicalization” OR “public policy” OR “public policy (law)” OR “regulations” OR “retail stores” OR “state laws” OR “state regulation”
AND			
3. Quantitative Methods	analysis OR “before‐and‐after” OR causal OR counterfactual OR “difference‐in‐difference*” OR experiment* OR “fixed effect*” OR “instrumental variable*” OR longitudinal OR matching OR meta‐analy* OR model* OR “panel data” OR “propensity score” OR “propensity match*” OR quasi‐experiment* OR regress* OR “regression discontinuity” OR “repeated measures” OR RCT OR “synthetic control” OR “systematic review” OR “time‐series”	OR	“causal modeling” OR “causal models” OR “controlled before‐after studies” OR “controlled before and after studies” OR “data analysis” OR “data analysis, statistical” OR “econometric models” OR “fixed effects model” OR “granger causality test” OR “instrumental variables (statistics)” OR “instrumental variable estimation” OR “interrupted time series analysis” OR “longitudinal method” OR “longitudinal studies” OR “meta‐analysis” OR “panel analysis” OR “panel data” OR “policy analysis” OR “population‐based case control” OR “pretests posttests” OR “pretest‐posttest design” OR “propensity score” OR “propensity score matching” OR “statistical analysis” OR “statistical matching” OR “quantitative research” OR “quasi‐experimental studies” OR “regression” OR “regression analysis” OR “regression discontinuity design” OR “repeated measures” OR “repeated measures design” OR “time series” OR “time series analysis”

^a^ Listed terms will be searched in title, abstract, and keyword fields.

#### 3.4.1 Academic databases

We will search the following indexed bibliographic databases and systematic review registries for eligible studies:

– Academic Search Complete (EBSCO)
– APA PsycINFO (EBSCO)
– Applied Social Sciences Index and Abstracts (ProQuest)
– Campbell Library (Campbell Collaboration)
– CINAHL Plus (EBSCO)
– Cochrane Library (Wiley)
– Criminal Justice Abstracts (EBSCO)
– Criminal Justice Database (ProQuest)
– Dissertations & Theses A&I (ProQuest)
– EconLit (EBSCO)
– Embase (Elsevier)
– ERIC (EBSCO)
– Health Management/Administration Database (ProQuest)
– Health Source: Nursing/Academic Edition (EBSCO)
– International Bibliography of the Social Sciences (ProQuest)
– Legal Source (EBSCO)
– Medline (EBSCO)
– National Criminal Justice Reference Service (ProQuest)
– PAIS Index (ProQuest)
– PROSPERO (University of York)
– Research Library (ProQuest)
– ScienceDirect (Elsevier)
– Web of Science: Core Collection (Clarivate Analytics)

#### 3.4.2 Gray literature databases

Additionally, we will search the following gray literature databases for eligible studies:

– Don M. Gottfredson Library of Criminal Justice Gray Literature Database (https://njlaw.rutgers.edu/cj/gray/index.php)
– European Monitoring Centre for Drugs and Drug Addiction (https://www.emcdda.europa.eu/publications)
– International Society for the Study of Drug Policy Grey Literature Bibliography (https://www.issdp.org/bibliography-introduction/)
– IZA – Institute of Labor Economics (https://www.iza.org/publications)
– National Bureau of Economic Research Working Papers (https://www.nber.org/papers.html)
– RAND Corporation (https://www.rand.org/search/advanced-search.html)
– National Drug & Alcohol Research Centre (https://ndarc.med.unsw.edu.au/resources)
– Open Society Framework (https://osf.io/preprints/)
– RePEc (https://ideas.repec.org/)
– ScholarWorks (https://scholarworks.gsu.edu/)
– Social Sciences Research Network (https://papers.ssrn.com/sol3/DisplayAbstractSearch.cfm)

#### 3.4.3 Contacting researchers and other supplementary approaches

We will distribute copies of the preliminary EGM within our professional networks and to authors of included EGM studies to seek additional studies of potential relevance. Study authors will also search their personal bibliographic libraries for relevant studies. Finally, we will perform backward and forward citation searching of included studies.

### 3.5 Screening and selection of studies

Retrieved studies will be independently screened at both title‐abstract and full‐text levels by two members of the study team. Specific screening questions for title and abstract review include the following:

1. Does the study examine a cannabis liberalization law or policy that expands legal access to cannabis supply for personal consumption?
2. Does the study examine (i) an intermediate market/environmental or attitudinal/behavioral outcome or (ii) a final health, safety, or socioeconomic outcome?
3. Is the study (i) a systematic review or (ii) does it employ a randomized, quasi‐experimental, or nonrandomized controlled study design providing quantitative evidence of impact?

If both screeners answer “yes” to all three questions, then the study moves to full‐text screening. Screening conflicts will be resolved by reaching consensus through arbitration with a third member of the study team.

At the full‐text screening stage, studies will be read and reviewed to ensure they clearly report (quasi)experimental results examining the effect of cannabis liberalization policies on relevant intermediate and final outcomes. Studies that do not meet these criteria or are published in other languages will be excluded at this stage.

### 3.6 Data extraction, coding, and management

Full bibliographic information along with key population, intervention, comparison, and outcome dimensions will be extracted for each study. We will also code additional study characteristics, including country, data years, publication type, unit of analysis, study design, and study quality. A draft coding guide is included in Appendix A. The PI will facilitate quality control and pilot data extraction. Eligible studies will be independently coded by two members of the study team, with any conflicts arbitrated and resolved by a third member of the study team.

### 3.7 Quality appraisal

Primary studies and systematic reviews will be assessed for quality using appropriate instruments. Current guidance to researchers is to choose a tool that will provide meaningful appraisals of bias based on included study designs (Quigley et al., 2019). Thus, we will finalize the critical appraisal tool post‐screening. Preliminarily, we plan to assess risk of bias for primary studies using either ROBINS‐I (Sterne et al., 2016), RoBANS (Kim et al., 2013), or SMS (Sherman et al., 2002) for observational studies and RoB 2 for RCTs (Sterne et al., 2019). For systematic reviews, we will assess the risk of bias using AMSTAR 2 (Shea et al., 2017). All risk of bias instruments will be independently coded by two members of the study team, with any conflicts arbitrated and resolved by a third member of the study team.

## 4 ANALYSIS AND PRESENTATION

### 4.1 Unit of analysis

The unit of analysis is a systematic review or primary study, represented as a single entry within each cell of the EGM. When multiple primary studies are reported from the same research project or underlying data, these studies will be entered independently into the EGM. When there are multiple reports of a single study, the reports will be combined for presentation in the EGM.

### 4.2 Planned analyses

The EGM report will provide descriptive graphical and/or tabular summaries of included studies, accompanied by narrative descriptions of the evidence. The report structure will include background, methods, results, and discussion sections.

Descriptively, in addition to presenting a conceptual framework and PRISMA flow diagram, an online interactive will be presented with two main dimensions: interventions and outcomes. Additional map dimensions will include the number of primary studies indicated by relative bubble size within the cells of the map, color coding of data points to reflect critical appraisal of study quality, and filtering by key study characteristics. Static maps presented in a report or article will present this information as a basic intervention‐outcome matrix, with additional tables and graphs such as bar and pie charts describing characteristics of included studies. Key factors include the following:

- Intervention and specific policy provisions
- Intermediate and final outcomes
- Population subgroups
- Country
- Data years
- Study design
- Study quality/risk of bias

### 4.3 Presentation

Cannabis liberalization policies and associated health, safety, and economic outcomes are the primary dimensions of the EGM. The following characteristics will be coded from primary studies to allow the map to be interactively filtered: population subgroup, country, data years, study design, comparator, unit of analysis, and risk of bias.

## 5 STAKEHOLDER ENGAGEMENT

The conceptual framework and intervention‐outcome categories were developed through a consultative process. Specifically, we solicited feedback from selected researchers and other stakeholders in the areas of drug and cannabis policy from various organizations, including the International Society for the Study of Drug Policy (ISSDP), Global Cannabis Cultivation Research Consortium (GCCRC), RAND Drug Policy Research Center (DPRC), and European Monitoring Centre for Drugs and Drug Addiction (EMCDDA).

The EGM will be shared with stakeholders through a number of different avenues, including:

a) Online interactive platform (e.g., EPPI Reviewer, 3ie).
b) Legislative briefings hosted by the Brookings‐USC Schaeffer Initiative in Washington, DC.
c) Presentations of working drafts of the document at policy and practitioner conferences, including Lisbon Addictions Conference, ISSDP, NBER Summer Institute, and Association for Public Policy Analysis and Management (APPAM).
d) Podcasts, research briefs, and editorials produced by the authors and disseminated through their various institutions’ external affairs offices (e.g., Schaeffer Center, National Bureau of Economic Research, Georgia State University).
e) Direct conversation with researchers in government and policymakers, made possible through the investigators participation in workgroups and committees with ISSDP, World Health Organization (WHO), National Institute on Drug Abuse (NIDA), EMCDDA, and the UN Office on Drugs and Crime (UNODC).

## ROLES AND RESPONSIBILITIES

The three key competency areas for EGM development (content, methods, and information retrieval) will be managed by the research team as explained below.

Content: Eric L. Sevigny and Rosalie L. Pacula provide content area expertise in marijuana policy. Eric L. Sevigny, the PI, has published extensively on drug and cannabis policy and has led or is leading other systematic reviews in the drug policy arena (drug courts, drug enforcement). Rosalie L. Pacula is a veteran NIH Investigator with dozens of published studies in this policy space. She has coauthored a previous meta‐analysis of MMLs (Sarvet et al., 2018) and served on technical expert panels of the impacts of cannabis policies for NIDA and WHO.

EGM methods: AMA is a recognized expert in research synthesis and meta‐analysis. He has published systematic reviews in education and other fields as well as cutting‐edge papers on systematic review methods and statistical approaches. He is the primary advisor on study design and statistical analysis. Eric L. Sevigny and Rosalie L. Pacula also have experience leading and contributing to systematic reviews.

Information retrieval: Policy Studies Librarian La Loria Konata (LLK) at Georgia State University will provide information retrieval advice and expertise. Danye N. Medhin and Jared Greathouse will retrieve, screen, and code studies under the supervision of Eric L. Sevigny.

## SOURCES OF SUPPORT

The proposed EGM is supported by funding from the National Institute on Drug Abuse (NIH Award No.: R03DA046806).

## DECLARATIONS OF INTEREST

Both Sevigny and Pacula have published primary research in this policy area. A number of these studies will likely be included in the EGM. Pacula has also coauthored a previous meta‐analysis on medical marijuana laws and adolescent cannabis use in the United States (Sarvet et al., 2018), as well as published a book on *Cannabis Use and Dependence: Public Health and Public Policy* (Cambridge University Press, 2003). EGM authors of primary studies included in the map will not be involved with coding their own studies.

## PRELIMINARY TIMEFRAME

Approximate time period for submission of the EGM is December 2020.

## PLANS FOR UPDATING THE EGM

The EGM will be updated on a periodic basis through June 2021 when funding for the EGM and subsequent meta‐analysis is set to expire. Eric L. Sevigny and Rosalie L. Pacula have an interest in maintaining and updating the EGM. The frequency of future updates will be contingent upon available resources.

## Footnotes

^1^
The term “marijuana” is American vernacular for cannabis plant material. Because it is a term that is used in US state liberalization statutes, we use this term to describe US state policies. The more general term “cannabis” will be used in all other instances.

## APPENDIX A EGM CODING GUIDE

1. **Policies/provisions**
  Major Type of Law
  – Comprehensive medical cannabis law
  – Limited medical cannabis law
  – Recreational cannabis law
  – Decriminalization of cannabis cultivation
  – Industrial hemp law
  User Authorization Provisions
  – Registry/identification cards
  – Qualifying medical conditions
  Medical Professional Provisions
  – Professional certifications
  – Provider‐patient relationship
  Caregiver Provisions
  – Registry/identification cards
  – Home cultivation
  – Plant/possession limits
  – Caregiver qualifications
  – Caregiver‐patient relationship
  Personal Supply Provisions
  – Home cultivation
  – Plant/possession limits
  Social Supply Provisions
  – Growing cooperatives/collectives
  – Cannabis social clubs
  – Plant/possession limits
  Commercial Supply Provisions—Production
  – Commercial production
  – Registration/licensing
  – Zoning/density
  – Physical security
  – Workplace safety
  – Pesticide and fertilizer controls
  – Cannabis waste regulations
  – Production limits
  Commercial Supply Provisions—Retail
  – Dispensaries/retail outlets
  – Cannabis cafes
  – Delivery services
  – Mail order services
  – Registration/licensing
  – Zoning/density
  – Physical security
  – Sales limits
  Product Provisions
  – Product type restrictions
  – Testing requirements
  – Labeling/packaging
  – Product tracking
  Marketing and Advertising Provisions
  – Advertising regulations
  – Product display regulations
  Financial Provisions
  – Taxes
  – Banking regulations
  Policy Operationalization
  – Law or statute
  – legal practice/implementation
2. **Outcomes**
  Changes in Markets and Environments
  – Arrests
  – Prices
  – Potency
  – Diversion
  – Marketing/advertising
  – Outlet density
  – Product contamination
  – Other (specify)
  Changes in Attitudes and Behaviors
  – Attitudes toward substance use
  – Prevalence of substance use
  – Patterns and frequency of substance use
  – Modes of consumption
  – Other (specify)
  Public Health Impacts
  – Substance use disorders
  – Treatment admissions
  – Emergency care utilization
  – Overdose
  – Suicide
  – Mental health
  – Physical health
  – Neonatal/pediatric exposure
  – Other (specify)
  Public Safety Impacts
  – Crime and disorder
  – Organized crime
  – Driving under the influence (DUI)
  – Traffic accidents and fatalities
  – Workplace safety
  – Production‐related explosions and fires
  – Other (specify)
  Socioeconomic Impacts
  – Housing values
  – Educational attainment
  – Productivity
  – (Un)employment
  – Sick days
  – Industry jobs
  – GDP
  – Criminal justice costs
  – Healthcare costs
  – Tax revenues
  – Drug tourism
  – Environmental consequences
  – Other (specify)
3. **Study characteristics**
  Country (specify)
  Data Years (specify)
  Publication Type
  – Peer‐reviewed article
  – Technical report
  – Unpublished working paper
  – Thesis/dissertation
  – Other (specify)
  Comparison
  – “Business as usual”/prohibition
  – Medical cannabis law
  – Other (specify)
  Target Population
  – General population
  – Adults
  – Young adults
  – Youth
  – Patients
  – Students
  – Criminal justice involved
  – Pregnant women
  – Other (specify)
  Unit of analysis
  – Individuals
  – Events
  – Jurisdictions
4. **Study design**
  – Systematic review
  – Cohort/follow‐up
  – Controlled before‐after/differences‐in‐differences/fixed effects
  – Interrupted time series
  – Regression discontinuity design
  – Synthetic control method
  – Propensity score analysis/matching
  – Instrumental variables estimation
  – Other design (specify)
5. **Risk of bias assessment**
  Risk of bias assessment (primary studies) (e.g., Sterne et al., 2016)
  – Low
  – Moderate
  – Serious
  – Critical
  Confidence rating (systematic reviews) (Shea et al., 2017)
  – High
  – Moderate
  – Low
  – Critically low
